# Genome editing in the green alga *Chlamydomonas*: past, present practice and future prospects

**DOI:** 10.1111/tpj.70140

**Published:** 2025-04-05

**Authors:** Adrian P. Nievergelt

**Affiliations:** ^1^ Max Planck Institute of Molecular Cell Biology and Genetics Pfotenhauerstraße 108 Dresden 01307 Germany; ^2^ Max Planck Institute of Molecular Plant Physiology Am Mühlenberg 1 Potsdam 14476 Germany

**Keywords:** *Chlamydomonas*, gene editing, CRISPR/Cas, genetics

## Abstract

The green alga *Chlamydomonas* is an important and versatile model organism for research topics ranging from photosynthesis and metabolism, cilia, and basal bodies to cellular communication and the cellular cycle and is of significant interest for green bioengineering processes. The genome in this unicellular green alga is contained in 17 haploid chromosomes and codes for 16 883 protein coding genes. Functional genomics, as well as biotechnological applications, rely on the ability to remove, add, and change these genes in a controlled and efficient manner. In this review, the history of gene editing in *Chlamydomonas* is put in the context of the wider developments in genetics to demonstrate how many of the key developments to engineer these algae follow the global trends and the availability of technology. Building on this background, an overview of the state of the art in *Chlamydomonas* engineering is given, focusing primarily on the practical aspects while giving examples of recent applications. Commonly encountered *Chlamydomonas*‐specific challenges, recent developments, and community resources are presented, and finally, a comprehensive discussion on the emergence and evolution of CRISPR/Cas‐based precision gene editing is given. An outline of possible future paths for gene editing based on current global trends in genetic engineering and tools for gene editing is presented.

## Significance Statement

This review discusses genome engineering in *Chlamydomonas* against the background of the history of genetic engineering, gives an overview of the tools, and methods available to a genetic engineer today and outlines potential routes for the field in the near future.

## INTRODUCTION

Algae, a diverse grouping of eukaryotic species capable of photosynthesis, are a topic of significant interest in ecology, fundamental biology, and biotechnology. Among today's established algal models, the green alga *Chlamydomonas* takes a special place as the likely best described and most versatile algal model to date (Goodenough, 2023). A number of key features make this unicellular organism particularly interesting: The cells have two motile eukaryotic flagella or cilia for motility, contain a highly efficient chloroplast that uses a starch‐plate enclosed, phase‐separated RuBisCo organelle for carbon concentration (Mackinder et al., 2016), and a meiosis‐capable (Dutcher, 1995) haploid genome partitioned into 17 chromosomes (Merchant et al., 2007). Additionally, *Chlamydomonas* is able to grow on minimal nutrients and is able to produce all essential amino acids, phosphoglycerides and ether lipids (Popko, 2017), carbohydrates as well as pigments such as chlorophylls and carotenoids. Finally, the cells can be transformed to incorporate exogenous DNA into the nuclear genome as well as the mitochondria or the chloroplast (plastid). These properties, among others, make *Chlamydomonas* a widely used system across science and engineering. For example, the structure and function of cilia is highly conserved across the phylogeny which makes *Chlamydomonas* an excellent platform for cilia research (Geyer et al., 2022; Han et al., 2022; Ma et al., 2019; Pigino et al., 2012, 2013). Similarly, key photoactive elements like Channelrhodopsins (Nagel et al., 2003) and important components of the photosynthetic pathway (Caspy et al., 2021; Chua & Gillham, 1977; Gorman & Levine, 1965) have been first discovered in *Chlamydomonas*. In addition to these applications in fundamental biology, algae are becoming increasingly important as a biotechnological chassis in response to an increasing demand for sustainable, green processes. The foundation laid by decades of work with *Chlamydomonas*, resulting in a versatile kit of methods and strains (Neupert et al., 2020) have paved the way for utilizing the diverse metabolic pathways for producing high‐value products such as carotenoids (Perozeni et al., 2020), terpenoids (Gutiérrez et al., 2024) or bio‐hydrogen (Hippler & Khosravitabar, 2024; King et al., 2022; Melis et al., 2000). The common denominator to all of these applications is the need for genetic engineering. Even though specific requirements vary somewhat depending on the field, the ability to introduce, remove or change genes is at the core of all work with *Chlamydomonas*. Here, genetic engineering of *Chlamydomonas* is reviewed in three sections: First, the historical context of how we arrived at today's precision engineering methods is presented, followed by an overview of present‐day methods and applications, and finally, a plot for potential routes forward to extend the current already powerful toolkit for this beautiful model organism is suggested.

## THE HISTORY OF *CHLAMYDOMONAS* AS A REFLECTION OF GLOBAL TRENDS IN GENOMICS

**Figure 1:**
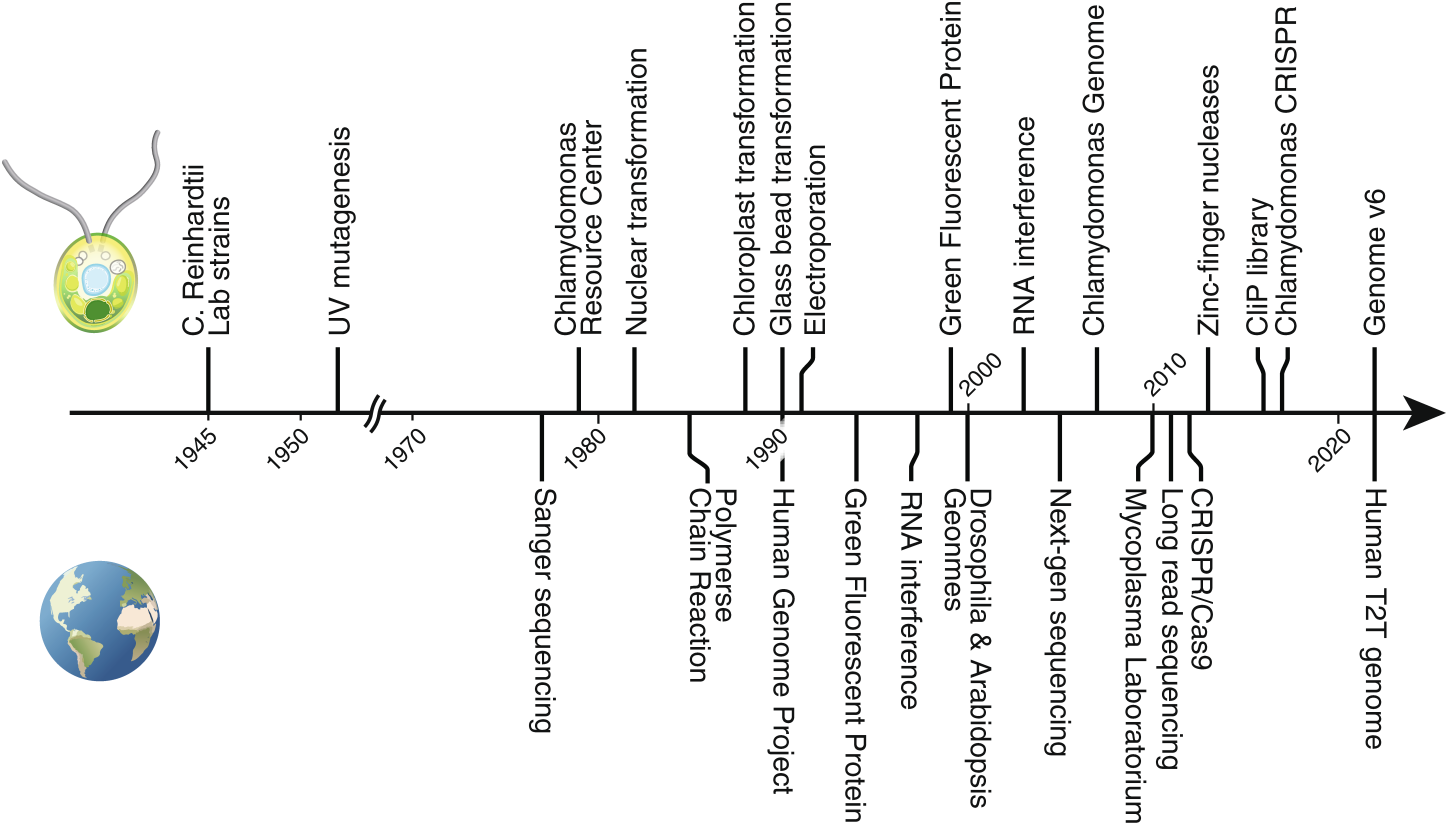
Non‐exhaustive timeline of key events in *Chlamydomonas* and global developments in genomics and genetic engineering.

*Chlamydomonas* is inherently a part of the global history of genetics and genetic engineering, and as such, the history of *Chlamydomonas* should be discussed against the background of the history of genomics in general (see Figure 1).

Although *Chlamydomonas* as a species was described much earlier, the strains used today originate from the progeny of a single zygospore isolated in a potato field in Massachusetts by Smith in 1945, resulting in four progeny cell lines that were diffused globally. How these lines moved from lab to lab is still subject to debate (Gallaher et al., 2015; Kubo et al., 2002). In the context of gene editing, it is of note that 137c (aka CC‐124/CC‐125), a strain deficient in nitrate assimilation, has emerged as the most widely used background strain. Early on, the DNA mutating effects of UV radiation were recognized and UV mutagenesis was successfully used in *Chlamydomonas* as one of the earliest methods for genetic manipulation (Lewin, 1952). Random mutagenesis and crossing had remained the only viable means of manipulating the *Chlamydomonas* genome for almost 40 years after the model was established, but together have been used to great effect in that time and remain relevant today (Neupert et al., 2009, 2020). In response to the rising popularity of the model, Gillham, Boynton, and Harris established a central species collection, later to become the *Chlamydomonas* Resource Center (CRC) which today is the primary enabler for freely sharing resources in the field. At the time, recombinant DNA technology was still in its infancy and routine sequencing, today an indispensable component of genetic engineering, only became widely available with the methods developed by Sanger (Sanger et al., 1977). In 1982, successful stable nuclear transformation of *Chlamydomonas* was demonstrated in Geneva by permanently rescuing an arginine auxotrophy mutant with an *arg4* gene from yeast (Rochaix & van Dillewijn, 1982), notably 3 years before polymerase chain reaction was first publicly disclosed (Saiki et al., 1985).

In those years, gene downregulation by antisense RNA notably became one of the earliest gene regulation tools in eukarya when the method was applied to carrot plants (Ecker & Davis, 1986). This advance was only applied in *Chlamydomonas* roughly a decade later (Schroda et al., 1999), shortly after the mechanism for gene silencing by double‐strand RNA interference (RNAi) was described in Nematodes (Fire et al., 1998), later awarded the Nobel prize. Half a decade later, this was reported in *Chlamydomonas* (Rohr et al., 2004) and has remained a versatile tool in *Chlamydomonas* work (Busch et al., 2008; Fei et al., 2023; Lechtreck & Witman, 2007; Moellering & Benning, 2010). Finally, microRNA‐based gene silencing has been shown in *Chlamydomonas* (Molnar et al., 2009; Zhao et al., 2009).

It was soon realized that *Chlamydomonas* integrates foreign DNA in essentially random locations of the genome, in contrast to, for example, yeast, which targets insertions based on sequence similarity (homology‐directed repair). A few years later, chloroplast transformation, which follows a very different biology, was achieved (Boynton et al., 1988). Shortly after, together with the beginning of the Human Genome Project, *Chlamydomonas* nuclear transformation through agitation with glass beads and electroporation resulted in much higher transformation efficiency than earlier methods (Kindle, 1990; Shimogawara et al., 1998) after which transformation was considered to be practical. In 1994, the first demonstration of using a green fluorescent protein fusion as a genetically encoded marker in the nematode *Caenorhabditis elegans* (Chalfie et al., 1994) revolutionized physiological imaging, but it took half a decade until this development was successfully translated to *Chlamydomonas* (Fuhrmann et al., 1999). In 2000, the publication of the fruit fly [*Drosophila melanogaster* (Adams et al., 2000)] and thale cress [*Arabidopsis thaliana* (The Arabidopsis Genome Initiative, 2000)] genomes arrived as harbingers of a series of revolutions in genomics, starting with the development of short‐read “next generation” sequencing in 2005 (Margulies et al., 2005). Just 2 years later, the *Chlamydomonas* genome of the cell wall deficient 137c strain *cw92*, sequenced with short‐read technology, was released (Merchant et al., 2007). This genome was a major step in facilitating *Chlamydomonas* genomics and genetic engineering. The years from 2010 to 2012 saw three key developments in genomics with significant implications for the work with *Chlamydomonas*: first, the fully synthetic bacterium *Mycoplasma laboratorium* (Gibson et al., 2010) marks the beginning of an era where synthetic *de novo* DNA is widely available and continually decreasing in price per basepair. Second, long‐read sequencing emerged, which overcomes many sequencing issues of the *Chlamydomonas* genome stemming from repeat regions and led to the telomere‐to‐telomere genome assembly [version 6 genome (Craig et al., 2023)] that has additionally been annotated with an excellent transcriptome by isoform sequencing. Finally, the discovery of the CRISPR/Cas system, an RNA programmable nuclease, marks the start of the era of precision genome engineering (Jinek et al., 2012). In *Chlamydomonas*, as in many other models, the only other option for targeted genetic edits at the time was zinc‐finger nucleases (Sizova et al., 2013), designer nucleases that have to be remade for every new desired target in the genome. Ironically, the fact that *Chlamydomonas* integrates DNA in an entirely non‐targeted manner has enabled the construction of genome‐wide mutant libraries in the Niyogi and Jonikas laboratories, which have now been established as powerful publicly shared resources (Dent et al., 2015; Li et al., 2016, 2019). The combined works of Greiner et al. (2017) and Ferenczi et al. (2017) and Picariello et al. (2020), each using different transformation strategies and repair template modalities, mark the start of the precision editing era of *Chlamydomonas*.

## THE GENOMIC TOOLKIT IS DIVERSE, POWERFUL, AND GROWING

### Commonly encountered challenges

Currently, *Chlamydomonas* genetic engineering is at a point where a significant portion of applications can be addressed with the available toolkit. In order to discuss present‐day developments, it is therefore relevant to review the common challenges that one would typically encounter.

Perhaps the most obvious of these is transformation efficiency: Empirically, *Chlamydomonas* seems particularly well equipped to defend against any kind of exogenous changes to its genome. UV mutagenesis for *Chlamydomonas* is frequently reported with much longer irradiation times than for other models like yeast (Neupert et al., 2009). This is perhaps not overly surprising as photosynthetic organisms are naturally exposed to UV radiation and *Chlamydomonas* in particular has no redundancy from a polyploid genome (Tokutsu et al., 2021). Likewise, the transformation rates with exogenous DNA are decidedly low. Glass bead transformation, achieving an efficiency of ~10^−5^, is considered a high‐efficiency method, a far stretch from what is routinely achieved in mammalian tissue culture systems. While the efficient repair and defence against foreign DNA in *Chlamydomonas* are interesting topics in their own right, they are nonetheless bad news for the genetic engineer.

Once cells have been transformed with exogenous DNA, they are typically selected with a dominant marker (de Carpentier et al., 2020; Meslet‐Cladière & Vallon, 2011; Sizova et al., 2001; Stevens et al., 1996; Yang et al., 2019). Expression of transgenes has been shown to vary significantly, both per isolated clone as well as over time. This is especially relevant for biotechnological applications which generally rely on high transgene expression and necessitate screening a significant number of colonies (Gutiérrez et al., 2022). It is not fully understood how transgenic expression levels in *Chlamydomonas* are determined at a mechanistic level, but it is generally accepted that a combination of codon optimization (Fuhrmann et al., 1999, 2004; Kwon et al., 2016) and strategic addition of introns (Baier et al., 2018, 2020) is non‐optional. An often‐overlooked factor for clone‐to‐clone variability of transgenic expression could relate to the fact that *Chlamydomonas* has a tendency to mutate exogenous DNA during insertion into the genome (Li et al., 2016; Nievergelt et al., 2023). Additionally, insertions at multiple loci of a single clone are common and can confound the elucidation of a phenotype. Finally, transgene expression tends to decrease over time due to gene silencing, most likely mediated by epigenetics (Cerutti et al., 1997; Neupert et al., 2020).

In a more practical aspect, working with *Chlamydomonas* genes commonly involves dealing with DNA of very high GC content (~64% average GC across the genome, with frequent stretches over 85%), repetitive regions, and occasionally very large proteins, further elongated by a large number of introns. The combination of these synergistic obstacles has limited the scope of genes that can reasonably be worked on to those of typically less than 10 kb, encompassing about 90% of the genome. This effect is further accentuated by the fact that only few *Chlamydomonas* promoters have been annotated, making it necessary to clone substantial extra sequence before the 5′ UTRs to increase the probability of including the endogenous promoters in the construct.

### Genomes and annotations

Unsurprisingly, in the context of genome editing, detailed knowledge of the makeup of the genome to be edited is key and the accuracy of the genomic assembly and annotation is crucial to successful genetic manipulation. Traditionally, these data have informed the sequences one needs to clone for a specific experiment. With modern precision genome editing, basepair accurate information on the entire genome is a requirement for selecting guide sites and ensuring their fidelity to prevent unintended off‐target edits.

Early work generally relied on sequencing single, small stretches of the genome, and the arrival of an annotated whole genome has had a dramatic impact on the possibilities available to the *Chlamydomonas* community in terms of genetics (Merchant et al., 2007). For all its strengths, this genome has also revealed some practical issues in the context of *Chlamydomonas* work, namely that mismatches between the digital reference genome and the physical genome of the current strain being worked arise from both technical limitations as well as a fairly high degree of biological variation throughout the different backgrounds present in the labs. It was later noticed that version 5 of the genome had both assembly errors due to repeat regions which cannot be covered with the short‐read sequencers which were the only technology available at the time. Additionally, there were non‐canonical gene‐chromosome associations caused by large‐scale rearrangements in the CC‐503 strain which was used for sequencing. This strain was chosen due to its similarity to the CC124/CC125 lineage and almost complete lack of cell wall, which significantly facilitates genomic DNA extraction, but it is now clear that either long‐term laboratory evolution or, more likely, the mutagenesis that caused the cell wall loss led to substantial additional genomic damage.

These issues were later addressed in the version 6 genome, which is based on long‐read sequencing technology that can accurately resolve long stretches of repeat regions (Craig et al., 2023). Notably, multiple long‐read‐based assemblies were made available for public use before. These include strains CC‐1690 (or 21gr), a highly resilient strain with biotechnological relevance sequenced by Oxford Nanopore (O'Donnell et al., 2020), an alternative, corrected CC‐503 assembly from PacBio HiFi reads (NCBI ASM1825784v1) and a high‐quality assembly of CC‐5816, a repeat back‐cross of the ubiquitous CC124 and CC125, from combined Oxford Nanopore and PacBio HiFi reads (Payne et al., 2023). Finally, PacBio‐based assemblies for the field isolates CC‐2931 (Chaux‐Jukic et al., 2021) and CC‐1952 (López‐Cortegano et al., 2023) are available. It has to be noted that, in contrast to the reference genome, these additional assemblies have been published without annotation, but are nonetheless a strong design resource and allow for an extended selection of background strains for editing. This selection will likely see a marked growth in light of the current efforts towards a *Chlamydomonas* pangenome (Chan & Salomé, 2023).

### Non‐targeted genome engineering

As targeted genome editing is a fairly recent development, the majority of genome engineering methods developed for *Chlamydomonas* are non‐targeting, that is, they do not act on an *a priori* known location in the genome. Despite their highly established status, these methods are still a fundamental pillar of genetic work in *Chlamydomonas* and continue to be actively developed, with the exception of UV mutagenesis, which has remained essentially unchanged for the last 70 years and does not need to be further discussed here.

The success of *Chlamydomonas* as a model for fundamental biology, like many other early model species, has relied primarily on collections of phenotypic mutants. This is reflected to the present day in the genetic nomenclature where many genes describe phenotypes. Examples are *pf* mutants which have paralyzed flagella (Yang et al., 2006), bld (read “bald”) mutants (Matsuura et al., 2004; Preble et al., 2001) which have no flagella or metabolic uptake and synthesis genes like the *arg* (needs arginine for growth) or *nit* (can't grow on nitrate) mutants (Auchincloss et al., 1999; Nichols & Syrett, 1978; Rochaix & van Dillewijn, 1982). Sixty years of these efforts have resulted in a large selection of, unfortunately, very heterogeneous strains that have resulted in many phenotype–genotype matches with many phenotypes still not genetically defined as of today. The large variation in genetic backgrounds has led the field to adopt a strict reliance on phenotypic rescue, where, in general, a phenotype–genotype relation is only accepted once a mutant has been rescued by insertional mutagenesis with a wild‐type version. Recently, the Jonikas lab has undertaken the effort of producing, maintaining, and sharing an insertional mutant library with two controlled genetic background strains of opposite mating types (Chlamydomonas Library Project, n.d.; Li et al., 2016, 2019). This transformative project is at present the default resource of mutant strains for the community and is an excellent example of how non‐targeted methods remain an important factor of *Chlamydomonas* genetics today.

Rescuing mutant phenotypes, as well as introducing transgenes requires the construction of donor DNA. These plasmids which are generally linearized for higher efficiency before transformation are composed of a promoter, a leader sequence (5′ UTR), a usually intron‐containing gene of interest coding sequence (CDS), and a terminator (3′ UTR). In most cases these plasmids also contain a dominant selective marker gene flanked by another promoter‐terminator pair which is required for selecting transformants. The most commonly used selection markers today confer antibiotics resistance to Zeocin, Spectinomycin, Hygromycin B, Paromomycin, Nourseothricin or Blasticidin S (Berthold et al., 2002; de Carpentier et al., 2020; Meslet‐Cladière & Vallon, 2011; Sizova et al., 2001; Stevens et al., 1996; Yang et al., 2019). Construction of these donor plasmids is now facilitated by the wide availability of synthetic genes and better PCR reagents (Nievergelt et al., 2023, 2024). The *Chlamydomonas* community also maintains an actively expanding modular cloning (MoClo) toolkit based on golden‐gate assembly that allows rapid construction of donor plasmids from a library of available parts such as promoters, UTRs, and reporter genes (Crozet et al., 2018).

### Targeted genome engineering

The ability to change a single specific location in the genome of an organism is an invaluable asset for genomic interrogation of model organisms, and *Chlamydomonas* is no exception. It is therefore all the more puzzling that the wide adoption of the CRISPR/Cas system (Jinek et al., 2012) in *Chlamydomonas* was surprisingly slow compared to many other organisms. From the original paper, it took 5 years for the first protocol with workable efficiency to be developed (Greiner et al., 2017), as opposed to about over a year for *Drosophila* (Gratz et al., 2013), *C. elegans* (Chen et al., 2013; Waaijers et al., 2013) or Zebrafish (Hwang et al., 2013; Irion et al., 2014), even though earlier attempts, which resulted in very low editing rates, were made (Jiang et al., 2014; Shin et al., 2016). Jiang et al. noted Cas9 toxicity as a possibility for the extremely low success rate, which Shin et al. addressed by using ribonucleoprotein (RNP) complexes instead, resulting in an increased success rate. A possible explanation for the initially discouraging results might be related to early work on CRISPR/Cas in other organisms, which showed that DNA damage from nuclease activity generally results in genomic scarring (small insertions and deletions) resulting from non‐homologous end joining (NHEJ) repair (Ran et al., 2013). This effect, which is commonly observed in most model organisms, is surprisingly difficult to reproduce in *Chlamydomonas*, most likely due to the presence of an exceedingly efficient NHEJ pathway combined with a generally low transformation efficiency. The pioneering work by Greiner proved the feasibility for precision genome editing, but practical efficiencies for insertional gene disruption were achieved only later by the Witman lab (Picariello et al., 2020). In turn, myself and other groups have extended this work to endogenous tagging and one‐shot multi‐locus integrations.

With the exception of scarring induced gene disruption, targeted genome editing in *Chlamydomonas* requires at least three components: A targeted nuclease and a nucleic acid donor and a method of delivery. Most recent work is distinguished by at least one of these components, which I will review here.

**Figure 2:**
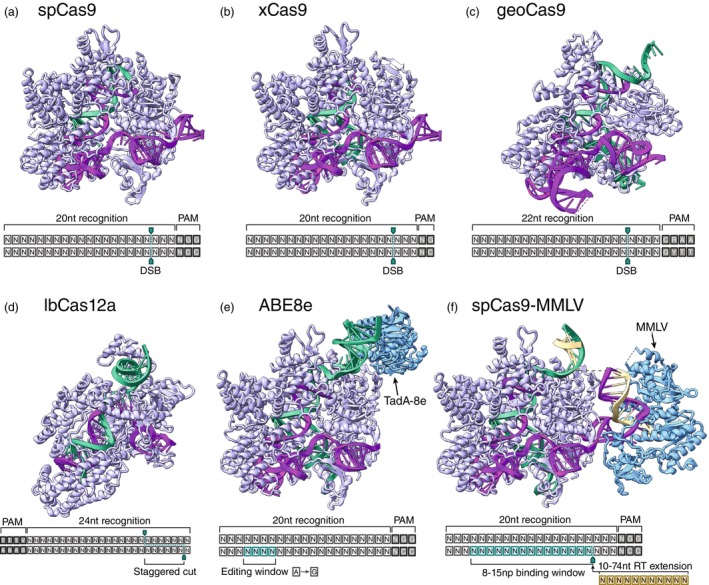
Different CRISPR enzymes extend the possibilities for genome editing. (a) The original *Streptococcus pyogenes* Cas9 recognizes a 20‐nucleotide long sequence followed by an NGG protospacer adjacent motif (PAM) and catalyzes a double‐strand break (DSB). PDB: 4OO8. (b) xCas9, engineered from spCas9, is structurally almost identical but allows for more permissible NG, GAA and GAT PAMs. PDB: 6AEG. (c) The highly stable and compact Cas9 homologue geoCas9 from *Geobacillus stearothermophilus* catalyzes a DSB with a CRAA PAM. PDB: 8UZA. (d) *Lachnospiraceae bacterium* Cas12a (Cpf1) is significantly smaller than Cas9 and produces a staggered cut with a TTTV PAM. PDB: 5XUT. (e) Base editors can catalyze single base mutations in a small window on the counter‐strand inside the recognition sequence. Shown as a representative example is the adenine base editor ABE8e catalyzing an A to G mutation via an engineered fused TadA enzyme. PDB: 6VPC. (f) Prime editors use a reverse transcriptase to cut‐and‐paste an RNA programmed DNA sequence of up to 74 bases at a single‐stranded nick on the opposing strand inside the recognition sequence. Shown here is the spCas9‐MMLV prime editor created as a fusion of an spCas9 nickase and a *Moloney Murine Leukaemia Virus* (MMLV) reverse transcriptase. PDB: 8WUT.

Before the advent of CRISPR/Cas, targeted nucleases have essentially been limited to zinc‐finger nucleases (ZNFs), artificial enzymes, usually based on the active site domain of the FokI that are designed to bind and cleave a 9–18 bp long DNA sequence by an array of specialized zinc‐finger domains (Kim et al., 1996). In practice, two complementary ZNFs have to be designed, expressed, and purified for each genomic target, which has hindered the wide adoption of ZNFs as genome editing tools beyond proof‐of‐concept studies, including in *Chlamydomonas* (Greiner et al., 2017; Sizova et al., 2013). In the larger genome editing field, transcription activator‐like effector (TALE) DNA binding domains (Boch et al., 2009) have become a popular alternative to ZNFs as designer nucleases, although this development has not been successfully translated to work in *Chlamydomonas* (Jiang & Weeks, 2017). With the timely arrival of the CRISPR/Cas gene editing platform in 2012, ZNFs have largely been replaced by RNA programmable nucleases since RNA is significantly easier to synthesize and purify than proteins. The majority of published genetic engineering in *Chlamydomonas* uses the Cas9 enzyme from *Streptococcus pyogenes* (See Figure 2a) with fused nuclear localization signals (NLS) as a primary nuclease (Chen, Yang, et al., 2023; Dhokane et al., 2020; Greiner et al., 2017; Hou et al., 2022; Kelterborn et al., 2022; Nievergelt et al., 2023, 2024; Picariello et al., 2020; Shin et al., 2016). Cas9 recognizes a 23 nucleotide long stretch of DNA where the last three bases are the required NGG protospacer adjacent motif (PAM) and catalyses a double‐strand break three bases upstream of this PAM (Jinek et al., 2012). The requirement for two consecutive guanine residues is perhaps one of the few times where the GC‐rich genome of *Chlamydomonas* is an advantage in practice. Early studies have explored the use of plasmid encoded Cas9 (Greiner et al., 2017; Jiang et al., 2014), but as with most other models, this has been widely replaced by *in‐vitro* pre‐assembled RNPs forming a fully programmed nuclease (Baek et al., 2016; Dhokane et al., 2020; Kelterborn et al., 2022; Shin et al., 2016). The set of published CRISPR effectors is rapidly expanding with variants on specificity, activity, stability, and PAM arriving at a rapid pace. Despite the potential benefits of these alternative enzymes, they have so far not been explored for use in *Chlamydomonas*, with the exception of Cas12a (Cpf1). Cas12a recognizes a TTTV (V = G/C/A) PAM and in contrast to Cas9 creates a staggered cut with 4–5 nucleotides in length (See Figure 2d). The single‐strand overhangs have the potential to increase homology‐directed repair (Ferenczi et al., 2017, 2021).

Successful precision edits in *Chlamydomonas* rely on the incorporation of donor DNA at the cut induced by the targeted nuclease. The use of both single stranded as well as double‐stranded donors has been extensively explored. Using single‐stranded oligodeoxynucleotide (ssODN) has been shown to potentially increase the efficiency of homology‐directed repair and may be easier to translocate into cells (Akella et al., 2021; Ferenczi et al., 2017, 2021; Sizova et al., 2021). On the contrary, long ssODNs, which are required for many functional tags, are more difficult to produce. Additionally, single‐stranded DNA will generally assume a complex secondary structure and this can mask the homology arms, which is a potential explanation for the very high variability that is observed for integration of ssODN donors in *Chlamydomonas* (Ferenczi et al., 2017). Conversely, double‐stranded DNA donors are routinely handled and can be produced directly from plasmid source using type IIs restriction digestion or by PCR amplification (Nievergelt et al., 2023; Picariello et al., 2020). In recent work, it has also been shown that purification quality is a major factor in successful precision genome editing (Nievergelt et al., 2023).

Independent of the nature of the nuclease and donor template, it has become clear that the method of how these are delivered into *Chlamydomonas* cells and how these cells were prepared is crucial for successful genetic edits. A key component of the bead transformation method introduced by Kindle is the removal of the tough cell wall with autolysin (Kindle, 1990). The early work by Shin and Greiner instead used the proprietary MAX Efficiency Transformation Reagent for Algae (Thermo Fisher, Waltham, MA, USA) which is a possible explanation for the low transformation efficiency of those works (Greiner et al., 2017; Shin et al., 2016). Notably, Greiner observed an increase in transformation efficiency and homology‐directed repair when cells were heat shocked at 40°C for 30 min immediately prior to transformation. This observation was confirmed by Picariello during a systematic optimization of the transformation procedure, which used autolysin‐based cell wall removal and achieved significantly higher efficiencies of targeted integration than previous works (Picariello et al., 2020). More recent work on endogenous tagging is in turn based on that optimization (Nievergelt et al., 2023, 2024). Notably, other factors have been shown to influence the efficiency of homology‐directed repair versus non‐homologous integration, including nitrogen starvation and cell cycle status (Angstenberger et al., 2020).

Delivery of RNPs and donor DNA into cells is typically achieved by electroporation. Glass bead transformation is not recommended for *Chlamydomonas* precision genome editing since it results in decreased efficiency (Picariello et al., 2020) and uses large reaction volumes (200–400 μl), and consequently significant amounts of nuclease. *Agrobacterium*‐mediated transformation has been used successfully, but does not appear to bring substantial benefits over electroporation (Mini et al., 2018). While many different electroporation setups have been successfully used in recent literature, there is some discussion as to which one yields optimal results. It is plausible that even traditional one‐pulse electroporators can work (Dhokane et al., 2020); it is generally accepted that more modern square‐wave electroporation yields better results (Hou et al., 2022; Nievergelt et al., 2023; Picariello et al., 2020). Based on the work by Greiner, the NEPA21 electroporator, a square‐wave electroporator that can deliver short high voltage “poration pulses” before the usual square pulses, has found widespread use in the field (Akella et al., 2021; Greiner et al., 2017; Takusagawa et al., 2021). It has been suggested that this poration pulse can replace cell wall removal in conventional transformation (Yamano et al., 2013). Most electroporators are cuvette based, thus typically need >100 μl reaction volume and suffer from inhomogeneous field effects leading to unwanted effects like arcing (Manicone et al., 2017). Recent work has demonstrated how a different electroporation geometry based on positive displacement pipettes can be used to downscale the reaction to just 10 μl while increasing the efficiency of the reaction, thus saving resources (Nievergelt et al., 2023, 2024).

With the wide adoption of precision genome editing, work on the fundamental biology of *Chlamydomonas* has now taken on an almost standardized workflow: For a gene of interest, mutants are sourced from the CLiP library, assayed for a phenotype, followed by recreating the phenotype in a line of choice by CRISPR/Cas mutagenesis and final rescue with a cloned insertion of the gene (Hwang et al., 2024; Viar et al., 2024). Given the broad interest in endogenous tagging, I expect to see this workflow extended by another step where the gene of interest is fused with a fluorescent and/or epitope tag for dynamic localization in life imaging (Hoepfner et al., 2024). The versatility of this technology has already been exemplified in highly creative work to probe ciliary microtubule post‐translational modifications by endogenously modifying the carboxy terminal tail of tubulin (Kubo et al., 2023).

### Chloroplast engineering

The chloroplast DNA of *Chlamydomonas* is contained in a circular plastid located in about 80 copies packaged to 5–10 nucleoids in the chloroplast (Kobayashi et al., 2017). In contrast to the nuclear genome, the chloroplast has only 34.6% GC content, codes for 72 proteins, and readily undergoes homologous recombination. As such, chloroplast engineering is distinctive from nuclear genome engineering and does not require targeted nucleases, although the concept has been explored (Yoo et al., 2020).

Chloroplast transformation is generally achieved by bombarding the cells with DNA coated nanoparticles, usually referred to as biolistic or gene‐gun transformation (Boynton et al., 1988). The primary challenge in Chloroplast editing is stability, as in, achieving a complete change of all ~80 copies of the plastome. This is usually done by consecutive sub‐plating on selective media and can be chemically assisted (Wurtz et al., 1979). Compared to nuclear engineering, chloroplast engineering has had fewer substantial improvements in methodology in recent years. Notably, a marker recycling system exists for chloroplast transformation, a possibility that is still under active development for the nuclear genome (Jackson et al., 2022). Recent work has leveraged high‐throughput workflows to develop a library of synthetic parts for chloroplast engineering including new regulatory elements (Inckemann et al., 2024), and inducible promoters (Rochaix et al., 2021) and temperature controlled elements (Chung et al., 2023) for the chloroplast have been previously demonstrated. Aside from pure methodology, there have been notable applications of chloroplast editing, including expression of full antibodies (Mayfield et al., 2003) or unlocking additional nutrient uptake pathways such as phosphite (Sandoval‐Vargas et al., 2018). Finally, a better understanding of the chloroplast structure, organisation and metabolic pathways is set to accelerate the use of chloroplast‐based processes in *Chlamydomonas* (Wang et al., 2023).

### Phenotypes, efficiency, sanity checks and the number of clones to keep

Arguably, genome editing should seldom, if ever, be seen in isolation. Rather it is generally seen as an enabling technology in the service of functional biology or biotechnological application. The tendency of *Chlamydomonas* to integrate DNA randomly, together with its haploid nature and the rather destructive nature of the traditional bead transformation process has previously resulted in phenotypes that were not directly related to a primary known mutation. Such phenotypes have occurred both from gene disruption due to the primary insert, secondary insertions as well as translocations or other large‐scale chromosomal rearrangements (Cariti et al., 2020; Craig et al., 2023; Li et al., 2019). Despite the increased control gained with precision genome editing, there is evidence that translocations can still occur, although it is currently unclear under which conditions and at what frequencies these occur (Payne et al., 2023). As such, the *Chlamydomonas* community has adopted a strict adherence to verification of phenotypes by meiosis and tetrad dissection (Dutcher, 1995) as well as phenotypic rescue of mutants with wild‐type genes (Debuchy et al., 1989; Diener et al., 1990; Kindle et al., 1989; Mayfield & Kindle, 1990). Until the advent of precision genome editing, this practice has been crucial and has prevented many erroneous reports. With the possibility to specifically target genes, this basic premise is generally adhered to, but the necessity to do so is subject to active discussion. The emergence of larger libraries (Cheng et al., 2017; Li et al., 2019) has resulted in the availability of multiple independent loss‐of‐function insertions for a single gene, which significantly increases the confidence of phenotype–genotype relations. With loss‐of‐function mutations created by precision genome editing one can typically isolate many independent clones with an identical primary insertion. As such, the question of how many of these independent lines should be retained is likewise subject to discussion.

To establish a rational argument to these questions in the context of precision genome editing, one has to estimate the frequency and nature of all different possible unintended genomic changes resulting from an editing experiment. The most obvious of these is secondary insertions of template DNA. The frequency of this can be estimated from whole‐genome sequencing, as well as the number of resistant colonies in a given experiment where the targeted locus is unaffected. With recent methods, the frequency of such secondary insertions is typically around 10–30% of resistant colonies (Nievergelt et al., 2023) and is likely to further decrease with future methods offering higher efficiency. The number of colonies per input as well as the number of correct resistant colonies are important measures for this and should likely be routinely reported. In case that secondary insertions are classical random insertions, the confirmation of the same phenotype in two independent colonies would almost guarantee a phenotype–genotype relation with the chance of both insertions disrupting the same gene being roughly the inverse of the number of total non‐lethal genes, less than 0.01%. Off‐target cuts in *Chlamydomonas* can in principle occur without careful guide design, but published data on off‐targets is relatively limited. The unusually efficient NHEJ repair pathway of *Chlamydomonas* most likely makes off‐target scars a non‐issue (Shin et al., 2016), with translocations or NHEJ mediated template integration being a much more likely outcome (Payne et al., 2023), but at present there is no statistical information available on the frequency of this occurring. As such, retaining two independent colonies is likely sufficient in most cases, while three or more are essentially a guarantee for a phenotype–genotype link in loss‐of‐function mutants. Complementation and crossing likewise are set to remain strong tools to confirm functional genomic links. Conversely, in *gain‐of‐function* mutants, fewer lines are often necessary to confidently establish these links. Especially for lines with fluorescently tagged proteins, previous knowledge about the protein and its likely location inside a cell typically makes it unnecessary to retain more than two isolates, with one generally being sufficient. This is particularly relevant for difficult loci with low efficiency where only a few successful edits are obtained in total.

Despite all of these estimations providing guidance for everyday lab work, the low cost and widespread availability of whole‐genome sequencing should likely be established as the gold standard for establishing a complete picture of the genome for functional genomics in *Chlamydomonas*.

## THE FUTURE OF *CHLAMYDOMONAS* ENGINEERING WILL REFLECT THE GENERAL PROGRESS IN GENE EDITING

The currently available toolkit for genome editing in *Chlamydomonas* is a versatile collection of community resources and methods that have helped to keep these algae as one of the most used photosynthetic organisms in research and biotechnology. I have outlined in this article how the *Chlamydomonas* community has leveraged the tools and methods developed by the wider genetics community to great success, all the while capitalizing on the properties unique to *Chlamydomonas*. It stands to reason that future work on *Chlamydomonas* genetics will keep following this trend.

As I have discussed here, the wider adoption of *Chlamydomonas* as a chassis for green bioprocesses has been somewhat limited by a number of factors, including gene silencing, poor transgene expression and a lack of understanding of genetic regulatory elements, and some of these have already been addressed in recent work (Baier et al., 2018; Beauchemin et al., 2024; Neupert et al., 2020). For example, the precise mechanism that requires *Chlamydomonas* genes to have introns, especially a first intron just a few tens of bases from the start codon, is the subject of current work. Understanding this mechanism is the most promising route towards creating new *Chlamydomonas* expression strains that can reach much higher transgene expression than is currently possible without the need for introns or endogenous regulatory elements.

Most genetic editing strategies to date rely on dominant markers for selection. The available number of these markers, however, is limited in number, which also directly puts a restriction on the number of genetic changes that can be made sequentially in a single line without crossing. Initial attempts using the Cre/LoxP recombination attempts showed successful excision of the cassettes but required an unwanted secondary transformation with a Cre recombinase expression construct (Kasai & Harayama, 2016). My own recent work has demonstrated how multi‐RNP co‐targeting strategies can be used to place the antibiotics marker on a locus separate from the primary edit, which can then be crossed out. This method, however, requires extensive screening and subsequent crossing steps and is likely most suited to applications in basic research (Nievergelt et al., 2023) or for creating engineered base strains for biotechnology. A cyclic auxotrophic marker system has recently been published (Ross et al., 2024), but to date, no robust marker excision system is widely used. Development of this system will require either a robust counterselection strategy to select for transformants with an excised resistance gene or, alternatively, a very high efficiency of excision to make selection unnecessary. An *E. coli* cytosine deaminase (codA) has been shown for counterselection in the chloroplast but has so far not been applied to the nucleus (Jackson et al., 2022).

Since the CRISPR/Cas system was established in 2012, a rapidly moving field has emerged around it with new variants and engineered CRISPR effectors being published at a rapid pace. While this stunning variety in detail is beyond the scope of this review, there are nonetheless important developments that are of possible interest for genome editing in *Chlamydomonas*. As discussed above, the only CRISPR enzymes that have presently been explored in *Chlamydomonas* are Cas9 and Cas12a (Cpf1). While the Cas9 PAM NGG is already well adapted to the GC‐rich *Chlamydomonas* genome, new variants have been engineered that offer more flexible choices of PAMs like the NG, GAA and GAT PAM sites of xCas9 shown in Figure 2(b) (Hu et al., 2018). Other variants have less permissive PAM sites but offer other benefits such as the compactness and high stability of geoCas9 shown in Figure 2(c) (Harrington et al., 2017) that recognizes a CRAA PAM (R = G/A). Importantly, the CRISPR system offers modes of action that go beyond double‐stranded breaks: The enzyme can be mutated to only bind a specific sequence without inherently modifying it, which allows genomically targeting other enzymes by fusing them to this “dead” dCas9. For example, a fusion of an adenosine deaminase (see Figure 2e) or a cytosine deaminase to a dCas9 results in base editors that can catalyze targeted single base A → G or C → T transitions (Gaudelli et al., 2017). Base editors are being continuously improved to achieve higher specificity, efficiency and flexibility (Chen, Zhang, et al., 2023; Huang et al., 2019), but have so far not been used in *Chlamydomonas*, despite their tremendous potential to be used for structurally guided functional genomics. Similarly, fusing a reverse transcriptase to a Cas9‐based nickase (single‐strand break inducer) results in an enzyme that can mediate RNA programmed “search‐and‐replace” insertions, called a prime editor, shown in Figure 2(f) (Anzalone et al., 2019). Like base editors, prime editors are being actively developed (Doman et al., 2023; Yan et al., 2024) and have not been harnessed for *Chlamydomonas* genetics. Since there is often a need for integration of larger fragments, CRISPR tools harnessing integrases (Yarnall et al., 2023) and transposases (Lampe et al., 2024) could be a potential future route to integration of larger fragments at higher efficiency in algal systems. Finally, there is a current rapidly growing interest in epigenetic regulation, and again, *Chlamydomonas* is likely set to be along for the ride (Beauchemin et al., 2024; Dai et al., 2024; Neupert et al., 2020). Among many other related up‐and‐coming developments, CRISPR effectors for targeted epigenetic editing have recently been demonstrated (Smith et al., 2022) and these tools offer promising options in conjunction with the precise clonal nature of *Chlamydomonas*.

In conclusion, there is a rapidly expanding genetic toolkit which is readily available that could be translated to work in *Chlamydomonas* and is likely to bring exciting new insights in the near future. In parallel, the *Chlamydomonas* specific resources will keep expanding and I expect that the members of our field will continue their exemplary practice of open collaboration and sharing of resources to extend the *Chlamydomonas* toolkit with new elements such as promoter and enhancer annotations (Sanabria et al., 2024), more versatile regulatory elements, expression strains and datasets to continue the success story of this beautiful model organism into the future (Boxes 1 and 2).

Box 1 **Bullet points**

- Gene editing in the green alga *Chlamydomonas* has been a reflection of global trends in genomics and tools for genetic editing.
- Highly established methods like UV mutagenesis and random insertional mutagenesis are still employed today, resulting in powerful community resources.
- Recent work has pioneered CRISPR/Cas‐based precision genome editing in *Chlamydomonas* for high‐efficiency targeted genome modifications.
- Future developments will likely harness the powerful genetic editing tools that are currently emerging at a high frequency, but applicability to *Chlamydomonas* will have to be assessed individually.

Box 2 **Open questions**

- What limits the integration of foreign DNA in *Chlamydomonas* and how can the integration efficiency be increased?
- How do introns mediate transgene integration, expression, and silencing, and can *de novo* introns be designed to leverage this control?
- What are the mechanistic differences that make the *Chlamydomonas* genome repair pathways so effective, and how could they be utilized?
- Could newer genetic tools such as base editors, prime editors or integrases with targeting be used for gene editing of the *Chlamydomonas* genome, plastome or mitogenome?

## CONFLICT OF INTEREST

The author declares no conflict of interest.

## Data Availability

Data sharing is not applicable to this article as no new data were created or analyzed in this study.
